# Use of Probiotics in Aquaculture

**DOI:** 10.5402/2012/916845

**Published:** 2012-10-16

**Authors:** Patricia Martínez Cruz, Ana L. Ibáñez, Oscar A. Monroy Hermosillo, Hugo C. Ramírez Saad

**Affiliations:** ^1^Departamento de Sistemas Biológicos, Universidad Autónoma Metropolitana-Xochimilco, Calzada del Hueso 1100, 04960 Mexico City, Mexico; ^2^Universidad Autónoma Metropolitana-Xochimilco, Calzada del Hueso 1100, 04960 Mexico City, Mexico; ^3^Departamento de Hidrobiología, Universidad Autónoma Metropolitana-Iztapalapa, Av. San Rafael Atlixco 186, 09340 Mexico City, Mexico; ^4^Departamento de Biotecnología, Universidad Autónoma Metropolitana-Iztapalapa, Av. San Rafael Atlixco 186, 09340 Mexico City, Mexico

## Abstract

The growth of aquaculture as an industry has accelerated over the past decades; this has resulted in environmental damages and low productivity of various crops. The need for increased disease resistance, growth of aquatic organisms, and feed efficiency has brought about the use of probiotics in aquaculture practices. The first application of probiotics occurred in 1986, to test their ability to increase growth of hydrobionts (organisms that live in water). Later, probiotics were used to improve water quality and control of bacterial infections. Nowadays, there is documented evidence that probiotics can improve the digestibility of nutrients, increase tolerance to stress, and encourage reproduction. Currently, there are commercial probiotic products prepared from various bacterial species such as *Bacillus* sp., *Lactobacillus* sp., *Enterococcus* sp., *Carnobacterium* sp., and the yeast *Saccharomyces cerevisiae* among others, and their use is regulated by careful management recommendations. The present paper shows the current knowledge of the use of probiotics in aquaculture, its antecedents, and safety measures to be carried out and discusses the prospects for study in this field.

## 1. Introduction

Aquaculture is the farming of aquatic organisms by intervention in the rearing process to enhance production and private ownership of the stock being cultivated. Compared to fishing, this activity allows a selective increase in the production of species used for human consumption, industry or sport fishing. Due to overfishing of wild populations, aquaculture has become an economic activity of great importance around the world. Aquaculture's contribution to world food production, raw materials for industrial and pharmaceutical use, and aquatic organisms for stocking or ornamental trade has increased dramatically in recent decades. The report World Aquaculture 2012 found that global production of fish from aquaculture grew more than 30 percent between 2006 and 2011, from 47.3 million tons to 63.6 million tons. It also forecasts that by 2012 more than 50 percent of the world's food fish consumption will come from aquaculture, so it is expected to overtaking capture fisheries as a source of edible fish. This growth rate is due to several factors: (1) many fisheries have reached their maximum sustainable exploitation, (2) consumer concerns about security and safety of their food, (3) the market demand for high-quality, healthy, low-calorie, and high-protein aquatic products, and (4) aquatic breeding makes only a minimum contribution to carbon dioxide emission [1, 2]. 

Aquaculture has a long history, originating at least in the year 475 B.C. in China [3], but became important in the late nineteen-forties, since the methods of aquaculture could be used to restock the waters as a complement to natural spawning. Nowadays, aquaculture is a lucrative industry [2, 4]. However, the intensification of aquaculture practices requires cultivation at high densities, which has caused significant damage to the environment due to discharges of concentrated organic wastes, that deplete dissolved oxygen in ponds, giving rise to toxic metabolites (such as hydrogen sulfide, methane, ammonia, and nitrites), that often are responsible for mortality. Additionally, aquaculture has appropriated of water bodies used for recreational purpose, and sometimes makes a water's waste because this natural resource is not reused in extensive aquaculture systems [5, 6]. Moreover, under these conditions of intensive production, aquatic species are subjected to high-stress conditions, increasing the incidence of diseases and causing a decrease in productivity [7]. 

Outbreaks of viral, bacterial, and fungal infections have caused devastating economic losses worldwide, that is, China reported disease-associated losses of $750 million in 1993, while India reported $210 million losses from 1995 to 1996. Added to this, significant stock mortality has been reported due to poor environmental conditions on farms, unbalanced nutrition, generation of toxins, and genetic factors [8]. In recent decades, prevention and control of animal diseases has focused on the use of chemical additives and veterinary medicines, especially antibiotics, which generate significant risks to public health by promoting the selection, propagation, and persistence of bacterial-resistant strains [9–11]. 

Resistance to antibiotics is acquired in two ways: chromosomal mutation or acquisition of plasmids. Chromosomal mutations are not transferred laterally to other bacteria but resistance plasmids can be transferred very rapidly, producing a high percentage of pathogenic bacteria that develop mediated plasmid-resistance in a short period of time [12]. This can be an important public health issue as well. For example, transfer of multidrug resistance occurred in Ecuador during the cholera epizootic happened between 1991 and 1994, which originated from shrimp farm workers. Although the original epidemic strain *Vibrio cholerae *01 was susceptible to 12 antimicrobial agents tested, on the coast of Ecuador it acquired multidrug resistance due to transfer of resistance genes from other *Vibrio* species that were pathogenic to shrimp [13]. 

Probiotic is a relatively new term which is used to name microorganisms that are associated with the beneficial effects for the host. Kozasa made the first empirical application of probiotics in aquaculture [14], considering the benefits exerted by the use of probiotics on humans and poultry. He used spores of *Bacillus toyoi *as feed additive to increase the growth rate of yellow tail, *Seriola quinqueradiata. *In 1991, Porubcan [15, 16] documented the use of *Bacillus *spp, to test its ability to increase productivity of *Penaeus monodon *farming and to improve water quality by decreasing the concentrations of ammonia and nitrite. In order to avoid or reduce the use of certain antimicrobials, biological control was tested, described as the use of natural enemies to reduce the damage caused by harmful organisms. Strictly speaking, a probiotic should not be classified as a biological control agent because it is not necessarily a natural enemy of the pathogen [17]. However, certain probiotics have the ability to inhibit the growth of pathogenic bacteria. Moriarty determined the ability of *Bacillus *spp. to decrease the proportion of *Vibrio *spp. in shrimp ponds, especially in sediments [18]. Further studies have stressed probiotics ability to stimulate appetite, improve absorption of nutrients, and strengthen the host immune system [19, 20]. The aim of this paper is to present an updated concept of probiotics, current uses in aquaculture, commercial presentations, and a final approach to prospect applications in this area. 

## 2. Definition of Probiotic

The term “probiotic” comes from Greek *pro* and *bios *meaning “prolife” [56], having different meanings over the years. In 1905, Dr. Elie Metchnikoff was the first to describe the positive role played by some bacteria among farmers who consumed pathogen-containing milk and that “reliance on gut microbes for food makes it possible to take steps to change the flora of our bodies and to replace harmful microbes by beneficial microbes” [57]. However, the term probiotic was introduced until 1965 by Lilly and Stillwell [58] as a modification of the original word “probiotika.” It was used to describe substances produced by a microorganism that prolong the logarithmic growth phase in other species. It was described as an agent which has the opposite function of antibiotics. Later, Sperti [59] modified the concept of “tissue extracts that stimulate microbial growth.” 

The first use of the term to describe a microbial feed/food supplement was by Parker in 1974 [60]. He defined it as “organisms and substances that contribute to intestinal microbial balance.” Fuller [61] expanded the definition to “live microbial food supplement that benefits the host (human or animal) by improving the microbial balance of the body” and said that it would be effective in a range of extreme temperatures and salinity variations. Afterwards, it was suggested that probiotics were “monocultures or mixed cultures of microorganisms applied to animals or humans, that benefit the host by improving properties of indigenous microflora” [62]. In 1998, Guarner and Schaafsma assumed that probiotics are live microorganisms which, when consumed in adequate amounts, confer health benefits to the host [63]. Gatesoupe in 1999, defined them as “microbial cells administered in a certain way, which reaches the gastrointestinal tract and remain alive with the aim of improving health” [25]. In the same year, studies were carried out on the inhibition of pathogens using probiotics, this expanded the definition to “… live microbial supplement which benefits the host by improving its microbial balance” [34]. 

Knowledge of probiotics has increased, currently it is known that these microorganisms have an antimicrobial effect through modifying the intestinal microbiota, secreting antibacterial substances (bacteriocins and organic acids), competing with pathogens to prevent their adhesion to the intestine, competing for nutrients necessary for pathogen survival, and producing an antitoxin effect. Probiotics are also capable of modulating the immune system, regulating allergic response of the body, and reducing proliferation of cancer in mammals. Because of this, when provided at certain concentration and viability, probiotics favorably affect host health [64]. In fact, terms such as “friendly bacteria,” “friendly,” or “healthy” are commonly used to describe probiotics [19]. 

For many years, studies focused on microorganisms characteristic from intestinal microbiota, and the term “probiotic” was mainly restricted to gram-positive lactic acid bacteria [65], particularly representative of the genera *Bifidobacterium, Lactobacillus, *and *Streptococcus *[66]. In contrast to terrestrial animals, gastrointestinal microbiota of aquatic species is particularly dependent on the external environment due to the flow of water passing through the digestive tract. Thus, the majority of bacteria are transient in the intestine, due to constant intake of water and food, together with microorganisms present in them. Although in the gastrointestinal tract (GIT) of aquatic animals have been reported potentially pathogenic bacteria such as *Salmonella, Listeria, *and *Escherichia coli*, probiotic bacteria and other microorganisms have also been identified. These include gram-positive bacteria such as *Bacillus, Carnobacterium, Enterococcus*, and several species of *Lactobacillus*; gram-negative, facultative anaerobic such as *Vibrio *and *Pseudomonas,* as well as certain fungi, yeasts, and algae of the genera *Debaryomyces, Saccharomyces, *and *Tetraselmis, *respectively [20, 67, 68]. Due to the increasing interest of probiotics in aquaculture, Moriarty [18] proposed extending the definition of these to “living microbial additives that benefit the health of hydrobionts and therefore increase productivity.” 

A more general and common concept of probiotic is “one or more microorganisms with beneficial effects for the host, able to persist in the digestive tract because of its tolerance to acid and bile salts” [20]. Although the use of probiotics in aquaculture is relatively recent, interest in them has increased due to their potential in disease control [19]; however, gradually other applications have been proposed (summarized in Table 1), that will be addressed in detail later. 

## 3. Commercial Preparations

The interest in probiotics as an environmentally friendly alternative is increasing and its application is both empirical and scientific. According to Soccol et al. [69], the global market for probiotic ingredients, supplements and foods, reached US $15,900 million in 2008 and is projected to increase to US $19,600 million in 2013, representing an annual growth rate of 4.3%. At present, there are several commercial preparations of probiotics that contain one or more live microorganisms, which have been introduced to improve the cultivation of aquatic organisms. Probiotics can be used as a food additive added directly to the culture tank or mixed with food. 

Apart from laboratory preparation of bacteria, some commercially available products are now available. One of the first evaluations of commercial products focussed on a bacterial preparation called Biostart that is derived from *Bacillus* isolates. It was used during the production of cultured catfish studying the effect of inoculum concentration [22]. In 1998, Moriarty reported that the use of commercial probiotic strains of *Bacillus *spp. increased the quality and viability of pond-raised shrimp [18]. Meanwhile, Chang and Liu [31] evaluated the effect of *Enterococcus faecium* SF68 and *Bacillus toyoi* isolates present in Cernivet LBC and Toyocerin, respectively, to decrease the mortality of the European eel because of the edwardsielosis, ensuring greater efficiency with *E. faecium *SF68. It is relevant to note that *E. faecium *has long been known as a probiotic for humans, whereas *B. toyoi *has been used with terrestrial animals. Moreover, a *B. subtilis* strain combined with hydrolytic enzymes to produce Biogen, was used to supplement the feed of *Oreochromis niloticus*, obtaining significant increases in productivity [70]. 

The lactic acid-producing bacteria have been the focus of much interest. The human probiotic, *Lactobacillus rhamnosus *ATCC (American Type Culture Collection, Rockville, MD, USA), was used in rainbow trout for 51 days to reduce mortality by *Aeromonas salmonicida, *the causative agent of the fish disease “furunculosis” (one of the major fish diseases in many parts of world). Mortality was reduced from 52.6 to 18.9% when 10^9^ cells g^−1^ were administered with feed, when probiotic dose was increased to 10^12^ cells g^−1^ of feed the mortality reached 46.3% [32]. Apparently, increasing dosage does not necessarily improve protection. Abasali and Mohamad [55] increased the gonadosomatic index and the production of fingerlings in females of reproductive age, using mixed cultures consisting of *L. acidophilus, L. casei, E. faecium, *and *B. thermophilum *(Primalac). 

Studies with *Penaeus vannamei *showed that using mixed cultures of probiotics increases survival, feed conversion, and the final production of farmed shrimp [71]. Meanwhile, Taoka et al. [72] used Alchem Poseidon and Alchem Korea CO and Wonju Korea CO, which have mixed cultures of bacteria (*Bacillus subtilis, Lactobacillus acidophilus, *and *Clostridium butyricum*) and yeast (*Saccharomyces cerevisiae*), enhanced nonspecific immune parameters of tilapia *Oreochromis niloticus* such as lysozyme activity, migration onf neutrophils, and plasma bactericidal activity, resulting in improvement of resistance to *Edwardsiella tarda* infection. Previous studies in humans and land animals using prebiotics (nondigestible ingredients of the diet that stimulates the growth of microorganisms) showed their ability to stimulate the activity of probiotic bacteria in the colon [73, 74]. Some commercial aquaculture products included prebiotics in their formulation, such as mannans, glucans, and yucca extract that further increase the beneficial effects of the product [75, 76]. 

Currently, commercial products are available in liquid or powder presentations, and various technologies have been developed for improvement. on the case of fermentation processes, the interest has been focused on optimizing the fermentation conditions to increase the viability and functionality of probiotics, improving performance [77]. Generally, the production is carried out in batch cultures due to the difficulty of industrial scale operation of continuous systems [69]. More recently, systems have been developed for immobilization of probiotics, especially using microencapsulation. Microbial cells at high density are encapsulated in a colloidal matrix using alginate, chitosan, carboxymethylcellulose, or pectin to physically and chemically protect the microorganisms. The methods commonly used for microencapsulation of probiotics are the emulsion, extrusion, spray drying, and adhesion to starch [78]. Focused on the application to aquaculture, Rosas et al. [79] have effectively encapsulated cells of *Shewanella putrefaciens *in calcium alginate, demonstrating the survival of encapsulated probiotic cells through the gastrointestinal tract of sole (*Solea senegalensis*). Encapsulation in alginate matrices protects bacteria from low pH and digestive enzymes; this protection helps to release the probiotic into the intestine without any significant damage [80]. Currently, the lyophilized commercial preparations have advantages for storage and transport. However, conditions for reconstitution of these preparations such as temperature, degree of hydration, and osmolarity of the solution are vital to ensure the viability of bacteria [81]. It is important to emphasize that these products must provide a health benefit to the host; for this, it is necessary that contained microorganisms have the ability to survive storage conditions, and after that in the digestive tract of aquatic species, remaining viable and stable, and finally improving production [20]. According to the opinion of the producers, these preparations are safe to use and effective in preserving the health of aquatic animals [19]. 

## 4. Applications of Probiotics in Aquaculture

The need for sustainable aquaculture has promoted research into the use of probiotics on aquatic organisms. The initial interest was focused on their use as growth promoters and to improve the health of animals; however, new areas have been found, such as their effect on reproduction or stress tolerance, although this requires a more scientific development. 

### 4.1. Growth Promoter

Probiotics have been used in aquaculture to increase the growth of cultivated species, in reality it is not known whether these products increase the appetite, or if, by their nature, improve digestibility. Some people are inclined to think that it could be both factors; furthermore, it would be important to determine whether probiotics actually taste good for aquaculture species [20]. According to Balcázar et al. [13], probiotic microorganisms are able to colonize gastrointestinal tract when administered over a long period of time because they have a higher multiplication rate than the rate of expulsion, so as probiotics constantly added to fish cultures, they adhere to the intestinal mucosa of them, developing and exercising their multiple benefits. This also depends on factors such as hydrobionts species, body temperature, enzyme levels, genetic resistance, and water quality. 

The effect of probiotics has been tested on phytoplankton (microalgae), which forms the basis of aquatic food chains, due to its nutrient-producing photosynthetic machinery that in most cases, higher organisms are unable to synthesize such is the case of polyunsaturated fatty acids and vitamins. Within groups of microalgae used in aquaculture are distinguished central diatoms as *Chaetoceros* spp., which have proven to be a good live food; however, production has limitations due to the complexity of their nutritional requirements [82]. Gómez et al. [83] assessed the growth of *Vibrio alginolyticus *C7b probiotic in the presence of the microalgae *Chaetoceros muelleri*, proving that these organisms can be grown together to achieve high density and fed to shrimp. 

Rotifers are indispensable as the first live feed for larvae of most cultured aquatic species, due to their small size they are more accessible to larvae, for example, the nauplii of brine shrimp, which is a very common live feed. Planas et al. [84] used lactic acid bacteria to increase the growth of the rotifer *Brachionus plicatilis* and obtained best results with the addition of *Lactococcus casei *ssp*. casei*, *Pediococcus acidilactici, *and *Lactobacillus lactis* ssp. *lactis.*

The use of probiotics as growth promoters of edible fishes has been reported. Diet of Nile tilapia (*Oreochromis niloticus*) was amended with a probiotic *Streptococcus *strain, increasing significantly the content of crude protein and crude lipid in the fish, also weight has increased from 0.154 g to 6.164 g in 9 weeks of culture [85]. Due to the commercial importance of this species, the effect of supplementing diet with probiotics produced an increase of 115.3% when commercial formulation was used at a concentration of 2% [70]. 

Examples of growth improvement of ornamental fishes include swordtail (*Xiphophorus helleri, X. maculatus*) and guppy, (*Poecilia reticulate, P. sphenops)*, their feed was supplemented with *Bacillus subtilis *and *Streptomyces*, finding significative increases in growth and survival of *Xiphophorus *and *Poecilia *after 90 and 50 days of administration, respectively [27, 86]. 

Probiotics also have been tested successfully in shellfish culture. Macey and Coyne [87] isolated two yeasts and one bacterial strain (designated SS1, AY1, and SY9, resp.) from the digestive tract of abalone (*Haliotis midae*). A diet was formulated with a mixture of the three putative probiotics. Each probiont was added to the feed to achieve a final concentration of approximately 10^7^ cells g^−1^ of dry feed. The growth rate of small (20 mm) and large (67 mm) abalone was improved by 8% and 34%, respectively, in eight-month cultures. Furthermore, abalones supplemented with probiotics had a survival rate of 62% to the pathogenic bacterium *Vibrio anguillarum *compared to 25% survival of untreated animals. 

### 4.2. Inhibition of Pathogens

Antibiotics were used for a long time in aquaculture to prevent diseases in the crop. However, this caused various problems such as the presence of antibiotic residues in animal tissues, the generation of bacterial resistance mechanisms, as well as an imbalance in the gastrointestinal microbiota of aquatic species, which affected their health [88]. In fact, the European Union has regulated the use of antibiotics in organisms for human consumption [89]. Today, consumers demand natural products, free of additives such as antibiotics; moreover, there is a tendency for preventing diseases rather than treating them. Thus, the use of probiotics is a viable alternative for the inhibition of pathogens and disease control in aquaculture species. 

Probiotic microorganisms have the ability to release chemical substances with bactericidal or bacteriostatic effect on pathogenic bacteria that are in the intestine of the host, thus constituting a barrier against the proliferation of opportunistic pathogens. In general, the antibacterial effect is due to one or more of the following factors: production of antibiotics, bacteriocins, siderophores, enzymes (lysozymes, proteases) and/or hydrogen peroxide, as well as alteration of the intestinal pH due to the generation of organic acids [65]. 

Taoka et al. [51] showed that viable probiotics administered to tilapia *Oreochromis niloticus*, increased non-specific immune response, determined by parameters such as lysozyme activity, neutrophile migration, and bactericidal activity, which improved the resistance of fish to infection by *Edwardsiella tarda*. In turn, Robertson et al. [90] isolated a strain of *Carnobacterium *sp. from salmon bowel and administered alive to rainbow trout and Atlantic salmon, demonstrating *in vitro *antagonism against known fish pathogens: *Aeromonas hydrophila, A. salmonicida, Flavobacterium psychrophilum, Photobacterium damselae, *and *Vibrio *species. There is also evidence on the effect of dead probiotic cultures consisting on a mixture of *Vibrio fluvialis *A3-47S, *Aeromonas hydrophila *A3-51, and *Carnobacterium *BA211, in the control of furunculosis in rainbow trout. For this specific case, the number of leukocytes was greater than with live cells, in fact, the data suggest that cellular immunity more than humoral factors was involved in the benefits of these preparations of inactivated bacterial cells [91]. In the case of shrimp, studies have focused on the evaluation of probiotics such as *Bacillus cereus, Paenibacillus polymyxa,* and *Pseudomonas* sp. PS-102 as biocontrol agents against pathogens of various *Vibrio *species [92, 93]. 

Probiotic strains isolated from the gastrointestinal tract of clownfish (*Amphiprion percula*) have been used to inactivate several pathogens such as *Aeromonas hydrophila *and *Vibrio alginolyticus* among others. It has been observed that probiotics *in vivo *generate a density such that allow the production of antimicrobial metabolites therefore, the bacteria isolated from adult clownfish have the potential to colonize the intestinal mucus and therefore can be used as prophylactic agent and/or therapeutic [94, 95]. Furthermore, it has been found that concentrations of 10^6^ to 10^8^ cells g^−1^ of probiotic promote the development of healthy microbiota in the gastrointestinal tract of ornamental fishes from the genera *Poecilia* and *Xiphophorus*, decreasing the amount of heterotrophic microorganisms [86]. 

Gómez et al. [96] reported the use of *Vibrio alginolyticus *strains as probiotics to increase survival and growth of white shrimp (*Litopenaeus vannamei*), also by using probiotics in Ecuadorian shrimp hatcheries, production increased by 35%, while with the use of antimicrobials it decreased by 94%. 

### 4.3. Improvement in Nutrient Digestion

A study has suggested that probiotics have a beneficial effect on the digestive processes of aquatic animals because probiotic strains synthesize extracellular enzymes such as proteases, amylases, and lipases as well as provide growth factors such as vitamins, fatty acids, and aminoacids [13]. Therefore, nutrients are absorbed more efficiently when the feed is supplemented with probiotics [70].

In this sense, probiotics have been used in edible fishes, as in the case of larvae of European bass (*Dicentrarchus labrax*). It has been reported that the probiotic yeast *Debaryomyces hansenii* HF1 has the ability to produce spermine and spermidine, two polyamines involved in the differentiation and maturation of the gastrointestinal tract in mammals. In addition this yeast secretes amylase and trypsin, enzymes that aid digestion in sea bass larvae [97]. Studies in juvenile common dentex *Dentex dentex* L. showed that when diet is supplemented with 0.5 g of *Bacillus cereus* strain E kg^−1^ of food, increased fish growth due to more efficient use of the food [98]. In the case of rainbow trout, similar results were obtained using *B. subtilis*, *B. licheniformis,* and *Enterococcus faecium*, when these probiotics were provided for 10 weeks along with the diet of fish [99]. In some trials, diet of European sea bass larvae (*Dicentrarchus labrax*) has supplemented with probiotic yeast (*Saccharomyces cerevisiae* strain X2180), assessing fish growth and activity and expression of antioxidative key enzymes (catalase, glutathione peroxidase, and superoxide dismutase), finding differences in enzyme activity and gene expression patterns between probiotic supplemented and nonsupplemented treatments, which was attributed to the presence of the yeast [100].

In white shrimp *Litopenaeus vannamei* Boone and *Fenneropenaeus indicus,* various strains of *Bacillus* have been used as probiotics to increase apparent digestibility of dry matter, crude protein, and phosphorus. Results showed higher sizes when the diet is supplemented with 50 g of probiotic kg^−1^ of food [101]. Other research has suggested the importance of managing the probiotic in all ontogenetic stages of the shrimp to generate a constant effect on the production of digestive enzymes [102].

In guppies (*Poecilia reticulata*, *P. sphenops*), and swordtail (*Xiphophorus helleri*, *X. maculatus*), the effect of incorporating *Bacillus subtilis*, isolated from the intestine of *Cirrhinus mrigala* into their diet has been evaluated. The results show an increase in the length and weight of the ornamental fishes as well as the specific activity of proteases and amylases in the digestive tract [86]. According to Moriarty [103], *Bacillus* secretes a wide range of exoenzymes that complement the activities of the fish and increases enzymatic digestion. In fact, the bacteria isolated from the digestive tract of aquatic animals have shown chitinases, proteases, cellulases, lipases, and trypsin [67].

### 4.4. Improvement of Water Quality

In several studies, water quality was recorded during the addition of probiotic strains especially of the gram-positive genus *Bacillus*. Probably since this bacterial group is more efficient than gram-negative in transforming organic matter to CO_2_. It is suggested that maintaining high levels of probiotics in production ponds, fish farmers can minimize the accumulation of dissolved and particulate organic carbon during the growing season. In addition, this can balance the production of phytoplankton [13]. However, this hypothesis could not be confirmed on tests carried out during cultivation of shrimps or channel catfish, using one or more species of *Bacillus*, *Nitrobacter*, *Pseudomonas*, *Enterobacter*, *Cellulomonas,* and *Rhodopseudomonas*. Thus published evidence for improving water quality is limited, except for the nitrification [65].

In the case of edible fish, trout production farms generate high concentrations of nitrogen ranging from 0.05–3.3 mg L^−1^ of total Kjeldahl nitrogen and up to 6.4 mg L^−1^ after 7 months of monitoring [104]. For tilapia production in recirculating systems, concentrations of total ammonia (NH_4_ + NH_3_) increased from 4.73 to 14.87 mg L^−1^ in a 21-day-experiment, while the nitrite concentration increased from 3.75 to 9.77 mg L^−1^ [105]. Due to the high concentrations of produced nitrogen compounds, especially the highly toxic total ammonia; the use of probiotics is recommended, as they may improve water quality. Haroun et al. supplemented the food of Nile tilapia *Oreochromis niloticus* L. with a commercial probiotic made from *Bacillus licheniformis* and *B. subtilis* in 17 weeks of culture. Assessment of water quality parameters showed an acceptable range for fish cultivation: 5.7–6.3 mg L^−1^ for dissolved oxygen concentration, 0.36–0.42 mg L^−1^ for ammonia concentration, and pH between 6.3 and 8.2 [70].

Lalloo et al. [106] isolated several strains of *Bacillus* from *Cyprinus carpio* and carried out tests to improve water quality in ornamental fish culture and to inhibit the growth of *Aeromonas hydrophila*. Three out of nine isolates showed high capacity to inhibit the pathogen in 78 of relative incidence rate; moreover, concentrations of ammonia, nitrate, and phosphate were lowered in rates of 74%, 76% and 72%, respectively. Contradictorily, Queiroz and Boyd [22] tested a commercial probiotic in catfish (*Ictalurus punctatus*), noting a survival and net fish production significantly higher when the probiotic was applied. However, very little differences were significant for the determined water quality variables (ammonia, chemical oxygen demand, nitrate, soluble reactive phosphorus, and dissolved oxygen) between the treated and control ponds. Taoka et al. [51] studied effects of commercial probiotics formulated from mixed cultures of bacteria and yeast on survival of Japanese flounder *Paralichthys olivaceus,* and water quality in a closed recirculating system. The probiotics-treated groups showed significantly greater survival rate as compared to the control group at the end of rearing experiment (50 days of culture), and water quality parameters were significantly lower in probiotics diet groups (from 0.24 ± 0.22 to 0.12 ± 0.10 mg L^−1^ of NH_4_, from 0.15 ± 0.08 to 0.08 ± 0.08 mg L^−1^ of NO_2_, and from 13.0 ± 3.9 to 10.2 ± 3.0 mg L^−1^ of PO_4_). Meanwhile, Wang et al. showed that a commercial product made from *Bacillus* sp., *Saccharomyces cerevisiae*, *Nitrosomonas* sp., and *Nitrobacter* sp. had the ability to increase the beneficial bacterial microbiota of *Penaeus vannamei* shrimp, further reducing the concentrations of inorganic nitrogen from 3.74 to 1.79 mg L^−1^ and phosphate from 0.1105 to 0.0364 mg L^−1^ [107].

### 4.5. Stress Tolerance

Aquaculture practices demand intensive productions in shorter times, causing stress in crop species. For example, it has been reported that chronic stress in zebra fish, *Danio rerio*, induces a general depression on the synthesis of muscle protein [108]. As a result, it was sought to increase stress tolerance by using probiotics. One of the firsts formal reports on this field studied the supplementation of *Lactobacillus delbrueckii* ssp. *delbrueckii* in the diet of European sea bass (*Dicentrarchus labrax*), at time intervals of 25 to 59 days. In addition to evaluating the growth improvement, hormone cortisol was quantified in fish tissue as stress marker, since it is directly involved in the animal's response to stress. Cortisol levels obtained in the treated fishes were significantly lower than those in the control (3.6 ± 0.36 ng g^−1^ and 5.1 ± 0.47 ng g^−1^, resp.) [49]. Another way to assess stress in fish involve subjecting them to heat shock, as in the case of Japanese flounder (*Paralichthys olivaceus*) grown in a recirculating system [72]. The stress tests were carried out until half the population died, thus calculated the mean lethal time (LT_50_) in the absence and with addition of a commercial probiotic containing *Bacillus subtilis*, *Lactobacillus acidophilus*, *Clostridium butyricum,* and *Saccharomyces cerevisiae*. The group treated with probiotics showed greater tolerance in the stress test than the control group, the LT_50_ was 40 and 25 min, respectively.

Lactate and plasma glucose levels are considered appropriate indicators of stress as they increase as a secondary response during periods of stress to cover high energy requirements induced by this situation. Therefore, Varela et al. have conducted studies on the gilt-head bream (*Sparus auratus*); the glycogen and triglycerides reserves in the livers of the control group were significantly decreased in relation to concentrations obtained when the fish feed was supplemented with the probiotic* Alteromonas* sp. Strain Pdp 11 [50].

Castex et al. [52] evaluated the effect of *Pediococcus acidilactici* MA 18/5 in the antioxidative response of the shrimp *Litopenaeus stylirostris* to oxidative stress. The results showed high activities of antioxidant enzymes; superoxide dismutase (283.7 and 153.7 mol min^−1^ in control and treated groups, resp.) and catalase (3.08 and 1.34 mol min^−1^ for the control and probiotic-treated, resp.).

The results obtained so far raise the possibility of preparing the fish in advance with probiotic treatment, for conventional aquacultures practices that create stress in animals, such as transport, change in water temperature, and periodic manipulations [46].

### 4.6. Effect on Reproduction of Aquatic Species

According to Izquierdo et al. [109], breeding aquaculture species have high nutritional requirements, thus reproductive capacity depends on appropriate concentrations of lipids, proteins, fatty acids, vitamins C and E, and carotenoids. Furthermore, the relationship of these components influences reproduction in various processes such as fertility, fertilization, birth and development of larvae. At present, for most cultured fish species, there are commercially available “broodstock diets” that just are larger-sized diets. In practice, many fish hatcheries improve the nutrition of their broodstock by feeding them solely on fresh fish byproducts or in combination with commercial diets. The most common fresh organisms used to feed broodstock fishes include squid, cuttlefish, mussels, krill, and small crustaceans. The use of these unprocessed fish products often do not provide adequate levels of nutrients needed by broodstock fishes, increasing the risk of pathogens transmission to the parents and offspring, including parasites, bacteria, and viruses. Therefore, probiotics added to food or water were used in order to prevent infections and to explore their effect on reproduction. 

The pioneer study on the effect of probiotic supplementation on reproductive performance of fish was carried out by Ghosh et al. [53], using a strain of *B. subtilis* isolated from intestine of *Cirrhinus mrigala*, incorporated at different concentrations to four species of ornamental fishes: *Poecilia reticulata, P. sphenops, Xiphophorus helleri,* and *X. maculates*, in a one-year experiment. The results showed that using *B. subtilis* concentrations of 10^6^–10^8^ cells g^−1^ of food, produced increases in the gonadosomatic index, fecundity, viability, and production of fry from the females of all four species. Furthermore, these authors proposed that complex B vitamins synthesized by the probiotic, especially thiamine (vitamin B1) and vitamin B12, contribute to reduce the number of dead or deformed alevins.

Abasali and Mohamad [55] carried out similar studies with *X. helleri*, using a commercial probiotic containing *Lactobacillus acidophilus*, *L. casei*, *Enterococcus faecium,* and *Bifidobacterium thermophilum*. The total production of alevin per female and the relative fecundity were evaluated. Their results showed significant differences between the control and probiotic-treated groups; in the first parameter 105 and 150 alevins were found on average, respectively. While in the second, 28 females were fecundated in the control and 41 for the probiotic treatment.

## 5. Safety Considerations of Probiotic

Traditionally, probiotics used in food industry have been deemed safe, in fact, no human risks have been determined, remaining as the best proof of its safety [110]. Theoretically, probiotics may be responsible for four types of side effects in susceptible individuals: systemic infections, deleterious metabolic activities, excessive immune stimulation, and gene transfer. However, no hard evidence has been found. 

In practice, there are few reports of bacteremia in humans, where isolation of probiotic bacteria from infections seems to be the result of an opportunistic infection caused by skin lesions, cancer, chronic illness, or a drug-induced abnormality. These conditions lead to a decreased intestinal barrier that promotes the passage of the bacteria through the mucosal epithelium. Subsequently, these microorganisms are transported to the mesenteric lymph nodes and other organs, leading to bacteremia that may progress to septicemia [111]. All reported cases of bacteremia occurred in patients with chronic illness or a weakened immune system [69, 112].

In the case of some lactic acid bacteria that are regarded as probiotics, the resistance to antibiotics can be linked to genes on chromosomes, plasmids, or transposons. However, there is insufficient information about the circumstances in which these genetic elements could be mobilized [113]. Moreover, it is recognized that some enterococci may possess virulence characteristics and have the ability to transfer antibiotic resistance elements, so it is recommended that no reference should be made to these organisms as probiotics for human consumption, unless the producer can demonstrate that this strain cannot acquire or transfer antibiotic resistance or induce infection [112].

Regarding safety of aquaculture products, in Asia and more recently in Latin America cultures of* Penaeus monodon* have been reported with bacterial white spot syndrome (BWSS), in farms with recurrent use of probiotics based on *Bacillus subtilis*. The spots are similar to those generated in the white spot viral syndrome (WSS), which is a deadly disease that spreads rapidly and causes mass mortalities in shrimp cultures [114, 115]. Wang et al. [116] demonstrated in 2000 that BWSS is a nonsystemic infection in *P. monodon* and lesions usually disappear after molting, under this condition cultures are still active and grow normally without significant mortality. However, it is of great concern because most farmers cannot distinguish BWSS from WSS. Farmers are advised in case of suspicion to send samples to the laboratory for confirmatory diagnosis.

Furthermore, since some aquaculture products are consumed raw or half cooked, has raised the question of whether residual probiotics may cause any infection in the final consumer. Shakibazadeh et al. [117] assessed the potential risk to humans caused by the use of probiotic *Shewanella algae* in shrimp farms. Studies were performed in mice that were given up to 10^36^ cfu to reach the LD_50_ value for *S. algae*, proving the safety of using probiotics in mice. Based on their results, these authors state that the use of *S. algae* is safe for the consumer of shrimps, such as for workers in the processing plants and farms.

Because there was no international consensus to ensure efficiency and safety of probiotics, FAO and WHO recognized the need to create guidelines for a systematic approach for the evaluation of probiotics in food, in order to substantiate their health claims. A working group with experts in the field was formed in order to recommend criteria and methodology for the evaluation of probiotics, based on scientific evidence [118]. As a result the “Guide for the Evaluation of Probiotics in Food” was presented, providing guidelines on the evaluation of health and nutrition properties of probiotics in food. The working group stated that no pathogenic or virulent properties were found in lactobacilli, bifidobacteria, or lactococci, although they acknowledged that under certain conditions, some strains of lactobacilli have been associated with rare cases of bacteremia. However, its incidence does not increase with raising the use of lactobacillus in probiotics. It was also mentioned that enterococci may possess virulence characteristics; therefore, it is not recommended as a probiotic for human consumption [113]. Although the guide is not focused on aquaculture products, it creates a precedent for conducting studies to evaluate the safety of probiotics in this area.

To date, the use of animal models including mice, rats, and fish has not revealed specific determinants of virulence or pathogenicity of the studied probiotic microorganisms, suggesting the overall safety of them [112]. However, it is important to continue research using three approaches: (i) analyzing the intrinsic properties of probiotic strains, (ii) studying its pharmacokinetics (survival, activity in the intestine, dose response, and recovery from mucosa), and (iii) understanding the interactions between the microorganism and the host [69].

## 6. Concluding Remarks

The current global food crisis and increasing production costs has put pressure on governments and the international community to ensure sufficient food supply for a growing population. Thus, aquaculture is presented as a way to meet the growing demand for fresh water food or seafood, and to meet current challenges relating to the ongoing globalization of trade, intensification and diversification of aquaculture, progress in technological innovations for food production, changes in ecological systems and human behavior, including a greater awareness to protect biodiversity, public health, and the environment. These challenges will lead to increased attention for improving aquaculture practices, and will become an important alternative to overexploitation and modification of aquatic ecosystems caused by capture fisheries. The use of probiotics can potentiate the benefits of this activity because, as presented in this paper, it offers viable alternatives for the generation of a higher-quality livestock product in terms of size, production time, and health.

In the near future, it is necessary to conduct studies relating to probiotics resistance to antibiotics, and the chances of transmission of genetic elements to other microorganisms in the fish GIT, and thus to humans when consuming the aquaculture product. 

On the other hand, there is a need to strengthen studies of microbial ecology in aquaculture systems, correlating microbial communities (microorganisms on water and in the GIT of aquatic species) with animal growth and its relationship to the water quality. Nowadays, a variety of probiotic strains present in the GIT of aquatic animals and nitrifying bacteria from biofilters have been isolated and characterized using biochemical, morphological, and molecular techniques [68, 119–121]. The development of molecular techniques such as PCR, FISH (fluorescent *in situ *hybridization), DGGE (denaturing gradient gel electrophoresis), and generation of genomic libraries have started to unveil the diversity present in aquaculture systems. Currently, next-generation sequencing methodologies offer great potential for phylogenetic identification of probiotic microorganisms without using conventional cultivation techniques.

## Figures and Tables

**Table 1:** Different applications of probiotics in aquaculture.

Application	Identity of the probiotic	Applied to aquatic species	Reference
Growth promoter	*Bacillus* sp. S11	*Penaeus monodon *	[21]
	*Bacillus* sp.	Catfish	[22]
	*Carnobacterium divergens *	*Gadus morhua *	[23]
	*Alteromonas* CA2	*Crassostrea gigas *	[24]
	*Lactobacillus helveticus *	*Scophthalmus maximus *	[25]
	*Lactobacillus lactis* AR21	*Brachionus plicatilis *	[26]
	*Streptococcus thermophilus *	*Scophthalmus maximus *	[25]
	*Streptomyces *	*Xiphophorus helleri *	[27]
	*L. casei *	*Poeciliopsis gracilis *	[28]
	*Bacillus* NL 110, *Vibrio* NE 17	*Macrobrachium rosenbergii *	[29]
	*Bacillus coagulans *	*Cyprinus carpio *koi	[30]

Pathogen inhibition	*Bacillus* sp.	Penaeids	[18]
	*Enterococcus faecium* SF 68	*Anguilla anguilla *	[31]
	*L. rhamnosus* ATCC53103	*Oncorhynchus mykiss *	[32]
	*Micrococcus luteus* A1-6	*Oncorhynchus mykiss *	[33]
	*Pseudomonas fluorescens *	*Oncorhynchus mykiss *	[34]
	*P. fluorescens *AH*2 *	*Oncorhynchus mykiss *	[35]
	*Pseudomonas*sp.	*Oncorhynchus mykiss *	[36]
	*Roseobacter *sp. BS. 107	Scallop larvae	[37]
	*Saccharomyces cerevisiae, S. exiguous, Phaffia rhodozyma *	*Litopenaeus vannamei *	[38]
	*Vibrio alginolyticus *	Salmonids	[39]
	*V. fluvialis *	*Oncorhynchus mykiss *	[33]
	*Tetraselmis suecica *	*Salmo salar *	[40]
	*Carnobacterium* sp. Hg4-03	*Hepialus gonggaensis *larvae	[41]
	*Lactobacillus acidophilus *	*Clarias gariepinus *	[42]
	*Bacillus *spp.,* Enterococcus*sp.	*Farfantepenaeus brasiliensis *	[43]
	*Lactococcus lactis *	*Epinephelus coioides *	[44]

Nutrient digestibility	*L. helveticus *	*Scophthalmus maximus *	[25]
	*Bacillus *NL 110,* Vibrio *NE 17	*Macrobrachium rosenbergii *	[29]
	*Carnobacterium *sp. Hg4-03	*Hepialus gonggaensis *larvae	[41]
	*Lactobacillus acidophilus *	*Clarias gariepinus *	[45]
	*Shewanella putrefaciens *Pdp11	*Solea senegalensis *	[46]

	*Bacillus* sp. 48	*Penaeus monodon *	[19]
	*Bacillus* NL 110, *Vibrio* sp. NE 17	*Macrobrachium rosenbergii *	[29]
Water quality	*Lactobacillus acidophilus *	*Clarias gariepinus *	[45]
	*B. coagulans* SC8168	*Pennaeus vannamei *	[47]
	*Bacillus *sp., *Saccharomyces *sp.	*Penaeus monodon *	[48]

	*Lactobacillus delbrueckii *	*Dicentrarchus labrax *	[49]
	*Alteromonas *sp.	*Sparus auratus *	[50]
Stress tolerance	*B. subtilis, L. acidophilus, S. cerevisiae *	*Paralichthys olivaceus *	[51]
	*L. casei *	*Poecilopsis gracilis *	[28]
	*Pediococcus acidilactici *	*Litopenaeus stylirostris *	[52]
	*Shewanella putrefaciens *Pdp11	Makimaki	[46]

Reproduction improvement	*Bacillus subtilis *	*Poecilia reticulata, Xiphophorus maculatus *	[53]
	*L. rhamnosus *	*Danio rerio *	[54]
	*L. acidophilus, L. casei, Enterococcus faecium, Bifidobacterium thermophilum *	*Xiphophorus helleri *	[55]
